# The Increase in Circulating Levels of Pro-Inflammatory Chemokines, Cytokines, and Complement C5 in Canines with Impaired Kidney Function

**DOI:** 10.3390/cimb44040114

**Published:** 2022-04-11

**Authors:** Selena K. Tavener, Dennis E. Jewell, Kiran S. Panickar

**Affiliations:** 1 Science & Technology Center, Hill’s Pet Nutrition, Inc., Topeka, KS 66617, USA; selena_tavener@hillspet.com; 2 Department of Grain Science & Industry, Kansas State University, Manhattan, KS 66506, USA; djewell@ksu.edu

**Keywords:** immune, renal, inflammation, PCR, histopathology

## Abstract

Chronic low-grade inflammation is a key contributor to the progression of kidney disease. The release of cytokines and other pro-inflammatory proteins may further contribute to detrimental kidney health by increasing interstitial edema and renal fibrosis. The aim of the present study was to investigate the inflammatory markers in canines who developed renal disease naturally and were diagnosed with renal disease either during life or following necropsy, as assessed by a veterinarian. RNA was isolated from canine blood obtained at necropsy and stored as bioarchived samples from ten canines with renal disease (9.6–14.7 yr) and ten controls (10.1–14.8 yr). At the time of death, the mean blood creatinine concentration and BUN were elevated in dogs with renal disease compared to control (both *p* < 0.01). Samples were assessed for changes in gene expression using the Canine cytokine RT^2^ Profiler PCR Array for inflammation. There was a significant increase in C-C Motif Chemokine Ligand 16 (CCL16), C-X-C Motif Chemokine Ligand 5 (CXCL5), Interleukin 16 (IL-16), and Complement Component 5 (C5) (all *p* < 0.05 vs. con). In addition, there was also a statistically non-significant increase in 49 genes and a down-regulation in 35 genes from a panel of total 84 genes. Pro-inflammatory genes including CCL16, CXCL5, IL-16, and C5 can all contribute to renal inflammation and fibrosis through different signaling pathways and may lead to a progressive impairment of kidney function. Blockade of their activation may be important in ameliorating the initiation and/or the progression of renal disease.

## 1. Introduction

Chronic kidney disease (CKD) is an irreversible and progressive loss of kidney function in companion animals including cats and dogs [1,2]. While a decline in nephron number and/or function is one contributing factor in kidney dysfunction, the causes of renal functional decline are likely multi-factorial. While urine specific gravity is one early indicator of kidney dysfunction, serum markers of kidney dysfunction generally include high levels of creatinine, blood urea nitrogen (BUN), and symmetric dimethylarginine (SDMA) [3,4].

Studies in rodents and humans have reported endothelial dysfunction, oxidative/nitrosative stress (OS/NS), and inflammation in the pathogenesis of impaired renal function [5,6,7,8]. It is unclear whether the precise molecular mechanisms that underlie kidney dysfunction are different in dogs when compared to humans or to rodent models of kidney dysfunction, but inflammation appears to play an important role in the decline in renal function in canines with CKD [9]. In addition, other risk factors in canines include advanced age, infectious diseases, prior anesthetic-surgical procedures, heart disease, neoplasms, endocrinopathies, and drugs that are nephrotoxic [10].

Inflammation has been reported to play an important role in the initiation and progression of reduced renal function [11]. Hasegawa et al. [12] described the role of cytokines including tumor necrosis factor-alpha (TNF-α), and interleukin-1 (IL-1) in the development of diabetic nephropathy in a rat model of kidney dysfunction. The causative factors of inflammation and OS/NS are not clear, but several factors, including modifications in lipid and lipoprotein metabolism [13,14], have been reported to increase inflammation. Uremic toxins in CKD including *p*-cresol and indoxyl sulfate [15] may also contribute to an increased inflammatory response [16]. Although there is an increase in pro-inflammatory cytokines in CKD, an interplay between pro- and anti-inflammatory cytokines including the target cells and/or the sequence of cytokine effect may also help explain the progression of CKD. Keepers et al. [17] reported monocyte chemoattractants including monocyte chemoattractant protein 1 (MCP-1/CCL2), macrophage inflammatory protein 1alpha (MIP-1α/CCL3), and RANTES (CCL5) in the kidney in a mouse model of hemolytic uremic syndrome. These cytokines highlight the role of inflammation in CKD and the possible role of uremic toxins in inflammation. There also appear to be previously unreported mechanisms associated with inflammation that are implicated in renal dysfunction, including the activation of NLR Family Pyrin Domain Containing 3 (NLRP3) inflammasome [18,19]. Suppression of pro-inflammatory markers and/or use of anti-inflammatory agents to reduce pathologies associated with CKD only further support the importance of inflammation in reduced renal function [20,21,22,23,24]. Thus, identifying and targeting newer inflammatory proteins in CKD may be an attractive strategy to alleviate the severity and progression of CKD.

In this retrospective study we assessed the gene expression of circulating pro-inflammatory markers, using a panel of 84 pro-inflammatory genes which also consisted of genes previously not assessed in renal dysfunction in canines using canines which had developed CKD naturally and were diagnosed with renal dysfunction either during life or following necropsy.

## 2. Materials and Methods

### 2.1. Experimental Design

This study was approved by the Institutional Animal Care and Use Committee, Hill’s Pet Nutrition, Inc., Topeka, KS, USA (Permit/approval Numbers: CP117, CP555, CP678, and CP836). Blood samples collected at end-of-life were used for assessment of gene expression in canines with CKD (*n* = 10; 9.6–14.7 yr) as well as controls (*n* = 10; 10.1–14.8 yr). Since this was a retrospective study, the blood samples for dogs with clinical diagnosis of CKD were assessed for biomarkers of renal dysfunction including serum creatinine, SDMA, and blood urea nitrogen (BUN). Collection of blood in RNA blood PAXgene tubes for gene expression as well as for Chem screen, CBC, and SDMA was conducted immediately prior to necropsy before euthanasia. The decision to euthanize was determined by the veterinarian, before pain and suffering took place and quality of life became poor. Histopathological analysis conducted by a veterinary pathologist also confirmed renal pathology including, but not limited to, glomerulosclerosis in these dogs. The control dogs were not reported to have signs of renal dysfunction. This study classified pets in the normal veterinarian-accepted fashion through the measurement of circulating concentrations of SDMA, urea, and creatinine as described by the International Renal Interest Society (IRIS) in 2019. However, as expected, it is normal to find some signs of renal decline in the kidney even in pets that do not meet the classification of having renal dysfunction. This histopathology study was conducted on tissues from pets and did not cause pain or hurt to any of the pets. In accordance to accepted veterinary practice all pets were cared for and supported during their life to optimize quality of life, even in the presence of renal disease. Samples were obtained in a postmortem examination to discover the cause of death or the extent of disease. If euthanasia was performed it was in order to stop the pets’ pain and suffering when it was concluded that the pets’ quality of life was degraded to a point where it was no longer humane to do otherwise. During life, all dogs were in group housing and all were allowed to exercise in outdoor play yards and also had access to toys.

### 2.2. Complete Blood Count (CBC)

Blood was collected immediately prior to necropsy for all dogs. The blood collected was analyzed for complete blood count and circulating concentrations of albumin, calcium, phosphorus, urea, and creatinine by an in-house laboratory (Roche Diagnostics, Cobas 6000 series, c501 module, Indianapolis, IN, USA). SDMA concentration was evaluated from banked samples (stored at −70 °C) by a commercial laboratory (IDEXX, Inc., North Grafton, MA, USA).

### 2.3. Histopathology

Tissue sections (5 μm) that were formalin-fixed and paraffin-embedded were used in the study for each dog. Hematoxylin and eosin-stained (H&E) renal cortex sections were examined under a microscope and assessed for changes in the morphology of each glomerulus in every selected field (29–34 fields/slide/dog) by an investigator who was blinded to the groups. A healthy glomerulus was defined as having morphologically well-defined basement membrane, mesangial cells, endothelial cells, podocytes, parietal epithelial cells, and Bowman’s capsule. In contrast, if a glomerulus showed morphological changes indicating disease, including but not limited to atrophy, no defined or significant thickening of the basement membrane, some expansion of the mesangial matrix or glomerular lesion, or mostly sclerotic, then the glomerulus was not considered ‘healthy’ [25]. The percentage of ‘healthy’ glomeruli was then calculated from the total number of glomeruli in each field, and an average of healthy glomeruli per field was obtained for every dog. Statistical analysis (*t*-test) was subsequently performed on the number of healthy glomeruli on the averages taken from all the control or CKD dogs.

### 2.4. Blood Collection and RNA Extraction

Gene expression was measured in blood (end-of-life samples) collected in PAXgene RNA blood tubes (Qiagen, Germantown, MD, USA) immediately prior to necropsy. Blood was stored as bioarchived samples after the dogs had lived their full lives until death. Total RNA was isolated using the PreAnalytix PAXgene blood RNA kit (Qiagen). The Agilent RNA 6000 Nano kit was used to measure the integrity of total RNA using the Agilent Bioanalyzer 2100 (Agilent, Santa Clara, CA, USA). The Qubit 3.0 Fluorometer was then used to measure the concentration of total RNA using the Qubit RNA BR Assay kit (Thermo Fisher, Waltham, MA, USA). cDNA synthesis was then performed using the RT^2^ First Strand kit (Qiagen).

### 2.5. Pathway-Focused Gene Expression Analysis

Changes in gene expression were analyzed using the Canine RT^2^ Profiler^TM^ PCR Array Dog Inflammatory Cytokines and Receptors array system (Qiagen) following the manufacturer’s instructions. Briefly, each 96-well array plate consisted of a focused panel of 84 genes. The last row of the array plate included the choice of five housekeeping genes for normalization, a specific well to detect genomic DNA contamination, reverse transcription control (RTC) wells to monitor the efficiency of the reverse transcription reaction, and positive PCR control (PPC) wells to test the efficiency of the PCR reaction. The total RNA input for each sample used for reverse transcription was 200 ng, and a component mix including cDNA reverse-transcribed from total RNA was prepared and loaded onto the plate. The cycling program was set to 95 °C for 10 min for the Hold Stage, followed by 40 cycles of 95 °C for 15 s and 60 °C for 60 s. A defaulted melting curve analysis was also performed to verify the specificity of the PCR run using the ViiA7 Real-Time PCR system (Thermo-Fisher). The C_T_ cutoff was set to 35 to be biologically meaningful. Data for the present study were normalized to HPRT1 as the housekeeping gene as HPRT1 showed the least amount of variance in Ct values in the control and CKD dogs.

### 2.6. Statistical Analysis

Where applicable, *t*-tests were used to compare means between groups. For the PCR array analysis, the raw CT values were exported to an Excel file to create a table of CT values. This table was then uploaded to the data analysis web portal at http://www.qiagen.com/geneglobe (accessed on 13 August 2020). Samples were assigned to controls and test groups. CT values were normalized based on a manual selection of reference gene (HPRT1). The ΔΔCt method was used for determining fold change with HPRT as the housekeeping gene. Fold changes for the PCR arrays were calculated relative to control, using the ΔΔCt method by normalizing to HPRT, a housekeeping gene included in each plate [26]. To analyze the relationship between age and mRNA, the values generated by calculating 2^−^^ΔCt^ were regressed against pet age at death.

## 3. Results

### 3.1. Complete Blood Count

**Figure 1:**
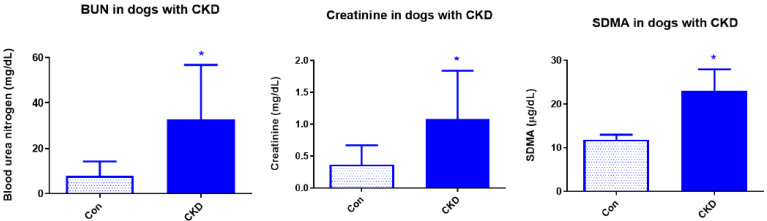
Circulating levels of kidney dysfunction. Increased levels of circulating biomarkers of renal dysfunction including creatinine, blood urea nitrogen (BUN), and symmetric dimethylarginine (SDMA) as assessed in the end-of-life samples. Data are presented as Mean ± SEM and BUN and creatinine (*n* = 10 Con; *n* = 10 CKD; * *p* < 0.05). For SDMA the data are also presented as Mean ± SEM but *n* = 5 (Con) and *n* = 4 (CKD; * *p* < 0.05).

This was a retrospective study, and the dogs were grouped into control and CKD based on the clinical diagnosis of the veterinarian during life. In addition, at the natural end-of-life, blood samples were collected from the control dogs as well as dogs with CKD. Biomarkers of renal dysfunction, including circulating levels of creatinine, blood urea nitrogen (BUN), and symmetric dimethylarginine (SDMA), were assessed in the end-of-life samples in order to identify and group the dogs into control and CKD groups. Creatinine and urea are nitrogenous end-products that are excreted by the kidney. There was a significant increase in creatinine, a product of muscle creatine activity that is removed by the kidneys, in the CKD dogs (1.84 ± 0.32 mg/dL; *n* = 10) when compared to controls (0.67 ± 0.05 mg/dL; *n* = 10; *p* ≤ 0.05; Figure 1), indicating reduced renal function in the CKD dogs. Urea is a by-product of protein catabolism and is predominantly cleared renally. Elevated level of circulating BUN is generally one of the accepted makers of impaired kidney function. There was a significant increase in BUN in the CKD dogs (56.74 ± 8.84 mg/dL; *n* = 10) when compared to controls (14.21 ± 1.45 mg/dL; *n* = 10; *p* < 0.01). SDMA, a sensitive marker of kidney function in dogs not influenced by lean body mass [3,27], is also elevated in dogs with CKD. SDMA values were not available in all dogs in the end-of-life samples (stored bioarchived samples). However, SDMA values were available for 4 out of 10 dogs with CKD and were significantly increased (23 + 4.9 μg/dL, *n* = 4) when compared to controls (11.81 ± 1.15 μg/dL; *n* = 5, *p* < 0.01).

### 3.2. Histopathology

**Figure 2:**
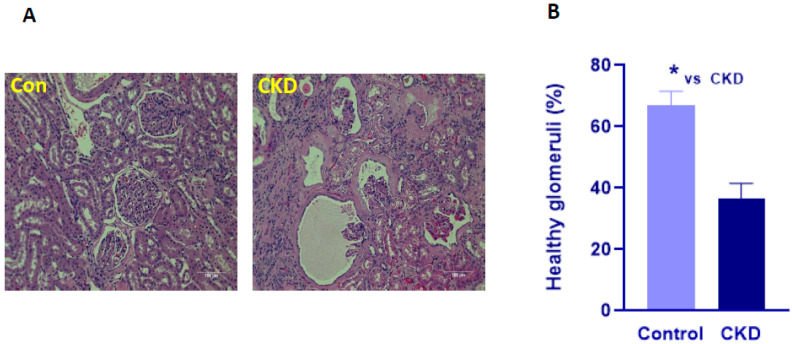
Photomicrographs of canine H&E- stained renal sections. (**A**) Representative photomicrographs of H&E-stained renal sections in Control (Con) and chronic kidney disease (CKD). Scale bar = 100 micron. (**B**) Quantification of healthy glomeruli per field in control (*n* = 10) and CKD dogs (*n* = 10). The number of healthy glomeruli was assessed in 29–34 fields in each slide for each dog (see text for details). Data are presented as Mean ± SEM (* *p* < 0.05).

To further characterize the dogs into control and CKD groups, we also examined the hemotoxylin-eosin (H&E)-stained renal sections for morphological differences in the renal corpuscle in all dogs. As mentioned above, 29–34 fields/dog/slide were examined. A healthy glomerulus was defined as having morphologically well-defined basement membrane, mesangial cells, endothelial cells, podocytes, parietal epithelial cells, and Bowman’s capsule. There was a significant decrease in the average number of healthy glomeruli in the CKD dogs (36 ± 5.01 %; *n* = 10) when compared to the control dogs (66.9 ± 4.5 %; *n* = 10; *p* < 0.05; Figure 2). Taken together with the circulating levels of markers of kidney dysfunction which differentiated the control dogs from the dogs with CKD, we assessed the differences in gene expression of circulating inflammatory markers in these dogs.

**Figure 3:**
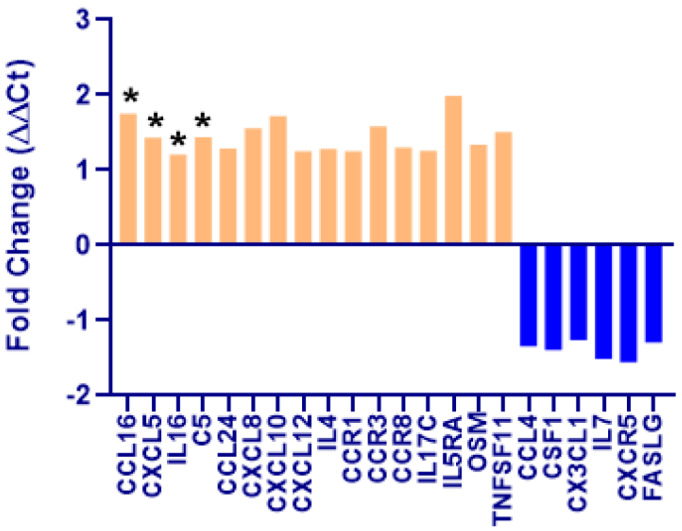
Graphical representation of selected genes that were up-regulated (orange) or down-regulated (blue) in dogs with chronic kidney disease (CKD; *n* = 10) when compared to controls (*n* = 10). There was a significant up-regulation in CCL16, CXCL5, IL-16, and C5 in CKD dogs compared to controls (* *p* < 0.05). Other genes that were up-regulated (>1.25 fold) are also shown, but they were not statistically significant. Down-regulated genes in CKD included CCL4, CSF1, CX3CL1, IL-7, CXCR5, and FASLG (>−1.25 fold; ns). Data are presented as mean fold change (ΔΔCt) normalized to HPRT1, the housekeeping gene. Fold-change values greater than one indicate an up-regulation. Fold-change values less than one indicate a negative or down-regulation, and the fold-regulation is the negative inverse of the fold-change (Qiagen).

Pathway-focused gene expression: We assessed the gene expression profile using the canine pathway-focused inflammatory PCR array panel. There was a statistically significant increase in 4 pro-inflammatory genes in CKD (*n* = 10) when compared to controls (*n* = 10). Chemokine ligand 16 (CCL16) increased 1.75-fold, C-X-C motif chemokine 5 (CXCL5) increased 1.43-fold, Interleukin 16 (IL-16) increased 1.21-fold, and complement 5 (C5) increased 1.44-fold in the CKD dogs when compared to the control dogs (all *p* < 0.05 vs. Con; Figure 3). In addition, the genes that were up-regulated ≥1.25-fold (arbitrary cutoff for the purpose of reporting only) but were not statistically significant included CCL24 (1.29), CXCL8 (1.56), CXCL10 (1.72), CXCL12 (1.25), IL-4 (1.28), CCR1 (1.25), CCR3 (1.58), CCR8 (1.30), IL-17C (1.26), IL-5RA (1.99), OSM (1.34), and TNFSF11 (1.51). In contrast, there were no genes that were down-regulated that were also statistically significant. Nevertheless, there were 6 genes that showed a greater than −1.25-fold decrease in the CKD dogs when compared to the control dogs including CCL4 (−1.34), CSF1 (−1.39), CX3CL1 (−1.26), IL-7 (−1.51), CXCR5 (−1.55), and FASLG (−1.29) (Figure 3). Further, the expression of six genes increased with age regardless of whether they were in control or CKD groups (CCL4, CCL5, CD70, IL17B, IL2RB, and FASLG (all *p* < 0.05)), and these genes did not have a reduction in expression associated with age (Table 1).

**Figure 4:**
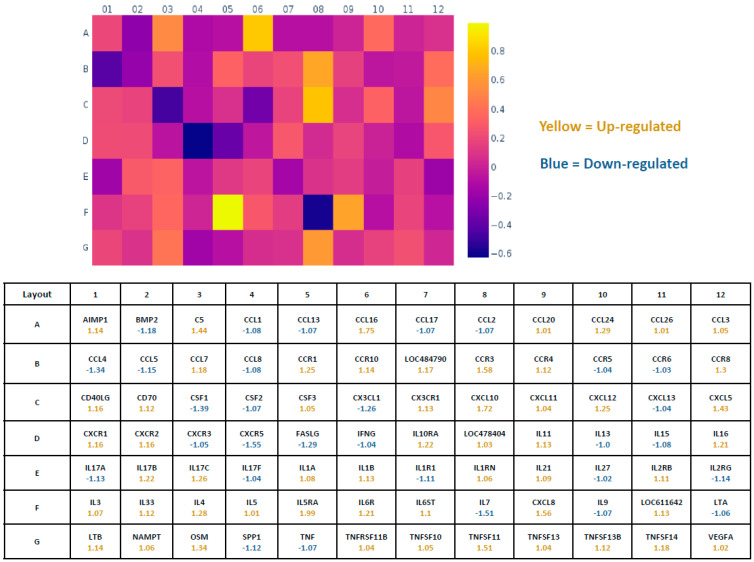
Heat map showing the fold change in gene expression in CKD dogs (*n* = 10) when compared to controls (*n* = 10). The individual squares in a heat map are scaled with a range of colors proportional to gene expression values. The figure below (Rows A to G) corresponds to the squares above, and each square indicates the average values for that gene, with numerically positive values indicating up-regulation and negative values indicating down-regulation. Row H (not shown) included the housekeeping genes ACTB, B2M, GAPDH, HPRT1, and RPLP in the assay. Other controls in row H included those for detection of genomic DNA contamination, reverse transcription control, and positive PCR control. As mentioned in Figure 3, data are presented as mean fold change (ΔΔCt) normalized to the housekeeping gene, HPRT1.

In general, when profiling the inflammatory gene expression in the CKD and the control dogs, there was an up-regulation in 49 genes and a down-regulation in 35 genes in the panel of total 84 genes (Figure 4) in CKD (*n* = 10) when compared to controls (*n* = 10). Fold-change values greater than one, indicated in the heat map (Figure 4), indicate a positive or up-regulation. However, several genes from the 49 up-regulated genes, including IL-5, CCL20, CCL26, CCL3, CSF3, CXCL11, IFNγ, TNFRSF11B, and TNFSF10, that showed a small increase in CKD (≤1.05 fold) when compared to controls, were not statistically different. Negative values indicate a down-regulation, where the fold-regulation is the negative inverse of the fold-change. Genes that showed a very small negative value (≤−1.05 fold), including CCR5, CCR6, CXCL13, IL-13, IL-17F, and IL-27, were unchanged in CKD when compared to controls.

## 4. Discussion

Our results show an increase in the gene expression of several pro-inflammatory chemokines and cytokines as well as complement activation in dogs with impaired kidney function when compared to controls. This is consistent with the role that inflammation plays in chronic renal dysfunction, including recruitment of T-cells, dendritic cells, and macrophages [28,29]. Interestingly, an increase observed in CCL16, CXCL5, and IL-16 in dogs with CKD in the present study is a novel observation since the role of these inflammatory genes has not been well-defined previously for their roles in CKD. CCL16 is a chemokine that acts on several receptors including CCR1 [30], CCR2, and H4 receptor [31], and thus may elicit multiple signaling pathways including the activation of monocytes, lymphocytes [32], and mast cells [33]. While an increase in the levels of CCL16 is associated with cardiovascular death in patients with cardiovascular dysfunction [34] and also with irritable bowel syndrome with diarrhea [35], its role in renal impairment is not clear. Further evidence supporting a detrimental effect for CCL16 in CKD comes from studies that investigated CCL16 receptors including CCR2. Sayyed et al. [36] reported that a CCR2 antagonist prevented glomerulosclerosis and renal failure in uninephrectomized type-2 diabetic db/db mice. Given that CCL16 is also a ligand for CCR2 receptor, it is conceivable that increased CCL16 plays a detrimental role in kidney function. In addition, blockade of CCR1, another receptor of CCL16 with a specific small molecule antagonist, has been reported to improve injury in several types of progressive kidney disease models [37]. Interestingly, we also observed a modest increase in CCR1 in the CKD dogs in the present study. Thus, the potential for CCL16 to act on various receptors on different cell types indicates that CCL16 may be exerting its effects through multiple signaling pathways. Blockade of CCL16 or its receptors may be important in attenuating the progression of renal dysfunction in CKD. It should, however, be noted that in contrast to the well-documented detrimental effect of increased concentration of CCL16 on renal function, low plasma CCL16 was significantly related to hepatic dysfunction in patients with chronic hepatitis B and liver cirrhosis [38]. The authors hypothesized that an increased CCL16 might reduce liver cirrhosis through inactivating hepatic stellate cells which, although beneficial in liver cirrhosis, would not be beneficial in attenuating the progression of renal disease. However, it is not clear whether this protective action of CCL16 is specific to the liver or applies more generally to the various stages of liver cirrhosis. Nevertheless, strategies to mitigate the downstream effects of CCL16 may be beneficial in reducing inflammation in CKD.

Interestingly, there was also an increase in selected cytokines and chemokines with age. This is not entirely surprising, as aging is associated with an increase in chronic low-grade inflammation [39]. However, our data indicate that the aging-associated increase in the selected cytokines was different from the inflammatory markers that were up-regulated in CKD when compared to controls. While it is likely that CKD is associated with several inflammatory pathways, it is conceivable that the aging-associated pro-inflammatory molecules may further contribute to the deleterious effects associated with the CKD-associated increase in cytokines. The precise relationship between differences in aging-associated and CKD-associated increase in pro-inflammatory genes is unclear and needs further investigation.

There was also an increase in other pro-inflammatory genes including CXCL5 and IL-16 in CKD in the present study. CXCL5 is a chemokine that is expressed by eosinophils and acts on the CXCR2 receptor [40]. While CXCL5 and/or CXCR2 are increased in patients with renal cell carcinoma [41], type-2 diabetic nephropathy [42], or acute rejection of kidney allograft [43], the role of CXCL5 in mediating continually declining kidney function is not clear. Our results provide a strong rationale for investigating further the role of CXCL5 in CKD. Similarly, the role of IL-16, a cytokine generally expressed in CD4^+^ and CD8^+^ T-cells, in impaired renal function is also not clear. Increased levels of IL-16, however, have been reported in patients with acute rejection of kidney transplantation [44] and in an ischemia-reperfusion model of injury in mice [45]. An et al. [46], while investigating the role of chronic inflammation in the development of diabetic nephropathy in macaque monkeys, reported decreased levels of IL-16 upon improving renal function therapeutically and reduced inflammation. These studies indicate the importance of strategies to reduce CXCL5 and IL-16 to exert a beneficial effect by reducing inflammation and likely slowing the progression of renal dysfunction.

Activation of the complement immune system and its role in renal pathophysiology has been well documented [47,48], including the role of C5 in renal inflammation in mice [49] and 3 glomerulopathy in a mouse model [50] and in renal fibrosis [51] (for review). Inhibiting the C5–C5a axis to reduce inflammation has been the target of a number of drug development studies in several human inflammatory conditions [52]. An anti-C5 monoclonal antibody, eculizumab, which binds to C5 and blocks its cleavage, is in clinical use in humans to reduce renal complement activation and renal injury [53]. Given the importance of the blockade of C5 pathway in reducing renal damage, it is clearly an important area of further investigation to study its role in reducing CKD and associated inflammation in dogs.

Several cytokines and chemokines were increased in CKD in the present study, including CCL24, CXCL8, CXCL10, CXCL12, IL-4, CCR1, CCR3, CCR8, IL-17C, IL-5RA, OSM, and TNFSF11, although they were not statistically significant. Nevertheless, these cytokines/chemokines and the receptors may contribute to the overall impairment in kidney function. For instance, Nentwig et al. [54] reported that dogs with impaired renal function had higher levels of circulating cytokines, including IL-1α, IL-1β, and TGF-β, as well as 5-lipoxygenase, an enzyme that catalyzes formation of inflammatory leukotrienes, when compared to controls. Interestingly, they further reported that while several of the dogs with acute kidney injury (AKI) had increased IL-8, none of the CKD dogs (*n* = 8) had higher IL-8 mRNA expression. Our study, however, shows an increased level of CXCL8 (IL-8) in dogs with CKD. The discrepancy of these findings is not clear, although their subjects were living during the course of the study whereas ours was a retrospective (end-of-life) study and so likely had dogs with different periods of naturally occurring CKD. We also saw a modest increase in the level of CCL24 (eotaxin-2), a pro-inflammatory chemokine, that acts on the receptor CCR3 and activates eosinophils [55]. Wang et al. [56] reported that serum levels of CCL24 were significantly elevated in patients with diabetic nephropathy and also in the kidneys in a mice model of diabetic nephropathy. However, when using the CRISPR-Cas9 technique to knock out CCL24 expression in podocytes in vitro, they reduced the podocyte inflammatory response that was artificially induced by high glucose, indicating a possible protective effect of CCL24. The role of eosinophil activation in naturally occurring renal disease is poorly understood. Whether increased serum CCL24 levels contribute to inflammation and reduced renal function in CKD is not clear, but it certainly warrants further investigation.

There was also a modest down-regulation of 35 genes in CKD when compared to controls, although only IL-7, CX3CL1, CXCR5, CSF1, and FASLG showed a greater than 1.25 down-regulation. Further investigation of cytokines and chemokines that are decreased is also important, as they may play a role in the overall initiation and progression of CKD. For instance, IL-7 is a cytokine growth factor for T and B-cells and inhibits fibrosis-like response in renal tubular epithelial cells in culture [57]. Whether a decline in IL7 may also promote renal fibrosis in canines is not clear. There are some discrepancies between our results and those reported in the literature, but it is not clear whether the differences are because of the differences in the regulation of inflammatory mechanisms in canines. Romanova et al. [58] has reported an increase in IL-7 protein levels assessed in the serum of patients of terminal-stage CKD. The complexity of the role of IL-7 in renal function in different species is still not understood. While blockade of IL-7 receptor improves long-term graft survival or graft tolerance in mouse kidney transplant models, the protective effect of blocking IL-7R, using anti-CD127 antibody, to improve transplantation was not observed in a kidney allograft model in baboons [59]. The role of IL-7 in naturally occurring CKD appears to be important and needs further investigation. Our study also shows a decreased level of CX3CL1 in CKD dogs. CX3CL1, a chemokine that acts on CX3CR1 receptor [60] and chemoattracts T-cells and monocytes, is up-regulated in response to pro-inflammatory signals [61]. However, CX3CL1 has also been hypothesized to play a role in cell survival as well as memory-associated synaptic plasticity in the brain [62]. Interestingly, the role of the CX3CL1-CX3CR1 axis has been shown to contribute to both detrimental and protective effects in various kidney diseases [61,63], and elucidating the diverse functions of CX3CL1 may be important in identifying therapeutic strategies to ameliorate renal inflammation and fibrosis. In short, the molecular changes that contribute to CKD are diverse, and the disease initiation and/or progression are conceivably influenced by up- as well as down-regulation of genes.

As reported in the review by Becker et al. [64] both canine and human chronic kidney disease have inflammation that leads to tissue damage and a subsequent loss of function through a loss of functioning nephrons. This increased inflammation is shown by an increased concentration of pro-inflammatory cytokines (either through enhanced production or reduced inactivation). As enhanced inflammation is directly proportional to decreased renal function, it is reasonable to investigate what inflammatory markers change in the presence of CKD. This paper suggests a possible understanding of such increased inflammation in the reported changes of inflammatory cytokines, and more importantly the cytokines/chemokines that need to be studied in future research. Further, the significance of the pathophysiological relation between CKD and cardiovascular disorders is increasingly recognized in dogs and cats with the aim of advancing the understanding of cardiovascular–renal disorders (CvRD) in pets [65].

This study has a primary limitation of a comparatively small sample size (*n* = 10/group) This may have caused true but small differences in response variables to be incorrectly concluded to be not different when a study with a larger n would have identified them. Because the classification was based on the presence or absence of CKD, some increased variation in response variables may have been introduced. A greater number of experimental subjects could allow for co-morbidity analysis, which was not performed in this study.

In summary, our study has identified several chemokines and their receptors that play an important role in CKD and very possibly in the development and progression of CKD. Some of the pro-inflammatory markers assessed in our study have not been investigated in the context of naturally occurring renal dysfunction and are novel observations that warrant further investigation. These data support future research into the cause of renal disease and the relationship with the subsequent development of these cytokine changes. Based on the findings from our study, it appears that the underlying mechanisms of CKD are multi-factorial. Increased levels of the pro-inflammatory chemokines and their receptors play an important role in the recruitment of T-cells, macrophages, and dendritic cells and subsequent inflammation.

## 5. Conclusions

Our data indicate that increased levels of circulating pro-inflammatory markers including CCL16, CXCL5, IL-16, and C5 could be considered good indicators of impaired renal function in dogs and warrant more investigation. The profile of circulating inflammatory markers observed in our study will also help in advancing our understanding of CKD as well as in developing strategies to ameliorate the initiation and/or progression in dogs with naturally occurring renal disease. Given that neutralizing antibodies to C5 are currently being used for reducing renal damage, targeted reduction in pro-inflammatory cytokines and chemokines may be attractive targets to reduce or attenuate the progression of kidney dysfunction.

## Figures and Tables

**Table 1:** The relationship of age and selected cytokine mRNA.

Cytokine ^1^	Calculated Amount at 10 Years of Age ^2^	Slope (Increase in Amount Per Year)	Standard Error	*p*-Value
CCL4	0.0236	0.0631	0.0221	0.01
CCL5	0.1241	0.1092	0.0426	0.02
CD70	0.0258	0.0074	0.0033	0.04
IL17B	0.0024	0.0005	0.0002	0.04
IL2RB	0.3335	0.1164	0.0442	0.02
FASLG	0.0271	0.0255	0.0081	0.01

^1^ Cytokines that had a relationship with animal age (*p* < 0.05). Cytokine data of all 20 animals were grouped to assess the age effect. ^2^ The value was calculated as 2^−ΔCt^, and number at age 10 was the intercept + slope × 10.

## Data Availability

Data are found in the paper or available from the corresponding author.
